# The impact of the COVID-19 pandemic on the rate of maternal postnatal healthcare examinations in England: an OpenSAFELY interrupted time series analysis providing evidence of disparity in care access

**DOI:** 10.1186/s12916-025-04436-w

**Published:** 2025-11-10

**Authors:** Dexter J. L. Hayes, Andrea L. Schaffer, Amir Mehrkar, Sebastian C. J. Bacon, Ben Goldacre, Brian MacKenna, Alexander E. P. Heazell, Tjeerd van Staa, Victoria Palin

**Affiliations:** 1 Maternal and Fetal Health Research Centre, Division of Developmental Biology and Medicine, The University of Manchester, Manchester, M13 9WL UK; 2 Bennett Institute for Applied Data Science, Nuffield Department of Primary Care Health Sciences, The University of Oxford, Oxford, OX26GG UK; 3 Centre for Health Informatics, School of Health Sciences, Faculty of Biology, Medicine, and Health, The University of Manchester, Manchester, M13 9PL UK

**Keywords:** Coronavirus, COVID-19, Maternal health, Pregnancy, Postnatal healthcare, Six-week check

## Abstract

**Background:**

Clinical guidance in England currently recommends that women undergo a postnatal health and wellbeing examination with a general practitioner 6-8 weeks after giving birth. The current study aimed to describe the impact of the COVID-19 pandemic on the rate of maternal postnatal examinations in England over time and its predictors, including socioeconomic deprivation and ethnicity.

**Methods:**

With the approval of NHS England, the OpenSAFELY platform was used to access the TPP SystmOne electronic health record (EHR) system for primary care. All records for registered female patients aged 14 to 49 years with a recorded birth coded between January 2019 and August 2023. Monthly rates of postnatal examinations were estimated based on the number of patients with a postnatal-related code within 6, 8 or 12 weeks of birth. Interrupted time-series analysis modelled the impact of the COVID-19 pandemic on the rate of examinations. Characteristics that may influence the likelihood of not having a postnatal examination were estimated using logistic regression.

**Results:**

For 626,180 patients with births coded, the rate of postnatal examinations increased with length of time after birth. Rates within 8 weeks fell from 368 to 279 per 1000 (↓24.1%) between January and March 2020, recovering to 402 per 1000 by January 2023. The incident rate ratio for the first national lockdown was 0.87 (95%CI 0.81–0.91) for 6 weeks, 0.84 (0.81–0.87) for 8 and 1.06 (1.04–1.08) for 12 weeks. The odds of no examination were affected by ethnicity (Asian or Asian British (OR 1.11, 1.09–1.14)), region (North East (1.39, 1.28–1.37) and West Midlands (1.33, 1.27–1.39)) and deprivation (most deprived (1.43, 1.31–1.37)).

**Conclusions:**

Maternal postnatal examinations within the recommended time were negatively affected by the onset of the pandemic. Despite rates improving over time, most failed to occur within the recommended 6–8 weeks. Significant variation in rates exists across NHS regions: rates were lower in ethnic minority groups and in more deprived populations. Addressing these disparities will require a combination of policy changes, financial incentives and targeted, culturally appropriate interventions to ensure equitable access to care for all mothers and infants.

**Supplementary Information:**

The online version contains supplementary material available at 10.1186/s12916-025-04436-w.

## Background

In England, the National Health Service (NHS) provides free healthcare, with midwives assessing mother and baby usually at least once within 36 h of birth and again at days 5 and 10, health visitors reviewing the baby’s health around days 7–14 and a postnatal assessment with a general practitioner (GP) (also known as a family doctor) at 6-8 weeks [1]. Recognising that a high quality postnatal maternal examination can impact both the short- and long-term health of mothers, the National Institute for Health and Care Excellence (NICE) recommends that this examination takes place 6-8 weeks after birth, as a separate appointment from the initial GP baby check and first vaccination appointments [2, 3]. This examination became a contractual requirement following an amendment to the GP contract in England, February 2020 [4].

The maternal postnatal examination should focus on recovery from birth, including both physical and mental wellbeing, and address any concerns, symptoms or signs of postnatal complications. The examination should also cover pregnancy-related conditions that may require ongoing care, identification of pelvic health issues, family planning and contraception [2, 5]. In addition to addressing immediate health risks, these appointments provide an opportunity to educate women on long-term risks (e.g. the risk of developing type 2 diabetes following gestational diabetes) and offer lifestyle interventions or additional clinical management to help minimise them.

A recent study examined the delivery of postnatal appointments in the UK, using data on approximately 34,300 births from the Clinical Practice Research Datalink between July 2015 and June 2018 [6]. Of these women, 62% had evidence of a face-to-face consultation indicating a structured postnatal review with a GP within 12 weeks of giving birth and just 40% of women had a structured review within the recommended 6–8 weeks. A further 27% had a record of one or more unclassified consultations. Women who experienced a preterm birth, were younger and registered at a practice in the most deprived areas were more likely to have a late, or no postnatal examination; this suggests that certain high-risk groups may not receive adequate postpartum care.

At the time of conducting our current study, there was little evidence of the impact of the COVID-19 pandemic on maternal healthcare interactions, particularly those occurring postnatally. In the beginning of the pandemic, advice from the NHS and UK government was not to go to GP surgeries, pharmacies, or hospital [7]. Guidance around COVID-19 and pregnancy at this time was uncertain due to a lack of evidence; further to this, reluctance to visit healthcare settings due to concerns about catching coronavirus was especially high in people who were pregnant [8]. From March 2020, a large proportion of primary care appointments in the UK changed from face to face appointments to an initial telephone or video call [9] and the number of in-person antenatal clinic visits decreased [10].

A systematic review of maternity care found a reduction in antenatal visits and an increase in remote care during the pandemic [11]. Furthermore, a survey of healthcare professionals at 194 obstetric units in England, conducted early in the pandemic, also reported a 70% and 56% reduction in routine antenatal and postnatal appointments, respectively, and over half the units used remote consultations in the postnatal period [12]. Whilst these changes reflected the pandemic’s impact on routine antenatal care, factors that increase the likelihood of having late or no postnatal examination overlap with many risk factors for contracting COVID-19, such as ethnic minority groups and deprivation [12].

A more recent study conducted across England (2006–2021) also identified persistent inequalities in the coverage of 6-8 week baby checks based on maternal ethnicity, even after adjusting for regional effects. These disparities varied by time and region, with women of Bangladeshi and Pakistani descent being disproportionately less likely to have a baby check, after accounting for sociodemographic factors, maternal and birth-related factors [13]. Although the study focused on infant examinations in primary care, it was observed that infants were less likely to receive a check if their mothers did not undergo a postnatal GP examination. This highlights the importance of maternal postnatal care in facilitating timely preventative care for both mothers and infants.

The current study aimed to evaluate the impact of the COVID-19 pandemic on the rate of maternal postnatal 6-8 week examinations, before, during and following the COVID-19 pandemic. This study also aimed to evaluate the association between maternal characteristics and the risk of no examination and how they might have been affected during the COVID-19 pandemic.

## Methods

### Data source

Primary care electronic health records (EHR) from patients registered within general practices managed by the software provider TPP SystmOne software were linked, stored and analysed securely using the OpenSAFELY platform [14], as part of the NHS England OpenSAFELY COVID-19 service. Data include pseudonymised data such as coded diagnoses, medications and physiological parameters. No free text data were included. All code is shared openly for review and re-use under the MIT open license https://github.com/opensafely/uom_pregnancy_tx_pathways. Detailed pseudonymised patient data is potentially re-identifiable and therefore not shared.

### Study population

A dynamic study population was generated by extracting monthly records for all registered women between 1^st^ January 2019 to 31^st^ August 2023 aged 14 to 49 years. Patients with missing sex and age were excluded and all live patients were required to have at least 1 year of continuous registration within the practice prior to the index date (the first date of each month).

### Codelists and data management

TPP SystmOne is fully compliant with the mandated NHS standard of SNOMED-CT clinical terminology. Clinical conditions and medicines are entered or prescribed in a format compliant with the NHS Dictionary of Medicines and Devices (dm+d) [15]. Pregnancy-related birth codes and codes referring to the postnatal 8-week period, as validated by the Clinical Practice Research Datalink (CPRD) [16], were converted to SNOMED CT using the NHS Technology Reference Update Distribution website [17] and refined by the study team and a clinical obstetrician so that only reliable delivery and postnatal codes were included. The final codelists are available at https://github.com/opensafely/uom_pregnancy_tx_pathways. Data management was performed using Python 3.8.10, with analysis carried out using R 4.0.5.

### Monthly measures

We adopted a similar approach to Li et al. and calculated the rate of postnatal examinations within 6, 8 or 12 weeks of codes relating to the birth of a baby, over time [6]. Specifically, for each monthly extract, if a birth-related code was present, the EHR record was searched to see if there was a postnatal code in the following 42, 56 or 84 days. Monthly rates were estimated by dividing the number of patients with a postnatal code in each follow-up period by the total number of patients with a birth code for each month, multiplied by 1000. All counts were rounded to the nearest 5, and counts less than 7 were redacted prior to calculating rates. To investigate whether postnatal examinations varied by maternal characteristics, rates were also estimated separately by age groups, region, deprivation quintile and ethnicity.

### Demographics

Time-varying patient demographics were extracted on each index date (first date of each month), including age, most recent body mass index (BMI) measurement to the index date and region of primary care practice. Records where a BMI was < 8 kg/m^2^ or > 50 kg/m^2^ were set to missing. Patient-level socioeconomic deprivation quintiles were estimated using the index of multiple deprivation (IMD) derived from the patient’s residence postcode within a Lower Super Output Area (LSOA) which comprises between 400 and 1200 households. Ethnicity was extracted once, recording the most recent entry. Common comorbidities were captured if recorded in primary care records for the 5 years before the delivery date and summarised as a weighted Charlson Comorbidity Index score [18]. Since patients could have multiple separate births across the study time frame, one random observation per patient was selected for each follow-up cohort and summarised using descriptive statistics.

### Statistical analysis

An interrupted time series (ITS) analysis assesses the impact of an intervention when implemented at a specific time period [19] and was used here to model the impact of the COVID-19 pandemic on the rate of postnatal examinations before and during the COVID-19 pandemic. Pre-COVID-19 was defined from 1^st^ January 2019 to 28^th^ February 2020. The start of the national lockdown period (March 2020) was modelled as the interruption in the ITS analysis. Negative binomial regressions were used to model the count of postnatal checks, with an offset for the population size for those with a birth-related code in 6, 8 or 12 weeks before (modelled separately). ITS models were adjusted for a binary variable to indicate COVID-19 time, a monthly count variable and time since the interruption variable. The time series counterfactual was calculated following the start of the pandemic to estimate what would have happened to the rate of postnatal check if there was no interruption by the COVID-19 pandemic. The incidence rate ratio (IRR) compares the ratio of rates between the two time periods. ITS was modelled overall and separately by age groups, region, deprivation quintile and ethnicity.

Regression analysis was used to investigate the association between maternal characteristics and the risk of having no record of a maternal postnatal healthcare examination. Again, since patients could have multiple separate births, one random observation from the initial cohort was selected for each follow-up cohort, and cohorts were modelled separately. The association was estimated by univariable and multivariable analysis adjusting for key maternal demographics with complete-case analysis. To model a change in the effect of maternal demographics on the odds of no postnatal examination before or during the pandemic, data was further stratified to 2019 or 2022 onwards, then modelled to compare any changes in odds ratios following the recovery.

## Results

### Description of the cohort

There was a total of 626,180 patients from 2535 practices in the study period with a recorded birth event. There were 96,280 (15.4%), 204,490 (32.7%) and 325,580 (50.0%) patients with a coded postnatal examination within 6-, 8- or 12-week follow-up of a delivery code, respectively. The mean number of clinical codes relating to the birth of a baby, per person, in the study period was 2.5 (SD = 2.0) for those with and 2.1 (SD = 1.6) for those without a postnatal examination within 12 weeks. Table 1 shows the characteristics of each of the study populations for the specified follow-up periods, with a mean age of 28 years at delivery, and the majority with a healthy BMI (36%), followed by overweight (23%) and obese (20–21%). A large proportion of the study population was White (~ 81%), followed by Asian (~ 11%) and Black (~ 3%) ethnicity with 0.4% missing. Most of the population had no comorbidities (~ 86%) as defined by the Charlson Index. The most common condition was asthma and/or chronic obstructive pulmonary disease (COPD) in 9.8% of the study population (see Additional file 1: Tab.S1).

**Table 1:** Study characteristics, randomly selecting one observation per patient for each follow-up period of the dynamic cohort. Counts are rounded to the nearest 5 and counts less than 7 redacted

		6-week cohort					8-week cohort					12-week cohort				
		Postnatal check					Postnatal check					Postnatal check				
		No		Yes		Total	No		Yes		Total	No		Yes		Total
Number of Delivery Codes	Mean (SD)	2.2 (1.7)		3.0 (2.2)			2.2 (1.7)		2.6 (2.1)			2.1 (1.6)		2.5 (2.0)		
Age	Mean (SD)	28.4 (5.7)		28.7 (5.6)			28.4 (5.8)		28.6 (5.5)			28.6 (5.8)		28.4 (5.6)		
Age Group	14–19	35,190	6.6	5655	5.9	40,845	29,190	6.9	11,655	5.7	40,845	20,400	6.8	20,445	6.3	40,845
	20–24	94,925	17.9	16,425	17.1	111,350	76,410	18.1	34,940	17.1	111,350	53,230	17.7	58,120	17.9	111,350
	25–29	170,195	32.1	30,950	32.1	201,145	134,250	31.8	66,895	32.7	201,145	94,075	31.3	107,070	32.9	201,145
	30–34	152,580	28.8	28,825	29.9	181,405	120,220	28.5	61,190	29.9	181,405	86,475	28.8	94,930	29.2	181,405
	35–39	64,990	12.3	12,345	12.8	77,330	51,770	12.3	25,560	12.5	77,330	38,485	12.8	38,845	11.9	77,330
	40–44	10,630	2.0	1935	2.0	12,565	8605	2.0	3960	1.9	12,565	6795	2.3	5770	1.8	12,565
	45–49	1395	0.3	145	0.2	1540	1255	0.3	285	0.1	1540	1145	0.4	395	0.1	1540
BMI	Mean (SD)	26.7 (6.1)		26.8 (6.1)			26.7 (6.1)		26.7 (6.0)			26.7 (6.1)		26.8 (6.0)		
BMI Group	Healthy weight	188,720	35.6	34,425	35.8	223,140	148,875	35.3	74,310	36.3	223,185	105,950	35.2	117,220	36.0	223,170
	Overweight	123,040	23.2	22,930	23.8	145,970	97,475	23.1	48,505	23.7	145,985	68,935	22.9	77,090	23.7	146,020
	Obese	110,950	20.9	21,010	21.8	131,960	88,030	20.9	43,880	21.5	131,910	62,015	20.6	69,920	21.5	131,935
	Underweight	13,940	2.6	2390	2.5	16,330	11,360	2.7	5000	2.4	16,360	8175	2.7	8190	2.5	16,365
	Missing	93,250	17.6	15,525	16.1	108,775	75,955	18.0	32,795	16.0	108,745	55,525	18.5	53,160	16.3	108,685
Region	East	122,670	23.2	22,625	4.3	145,290	91,890	21.8	53,390	10.1	145,280	60,460	11.4	84,830	16.0	145,290
	East Midlands	94,060	17.8	15,390	16.0	109,445	77,805	18.5	31,645	15.5	109,450	59,665	19.9	49,775	15.3	109,440
	London	28,485	5.4	7905	8.2	36,385	24,235	5.8	12,145	5.9	36,380	20,105	6.7	16,280	5.0	36,385
	North East	26,390	5.0	4160	4.3	30,550	22,410	5.3	8155	4.0	30,565	16,725	5.6	13,840	4.3	30,560
	North West	48,350	9.1	8995	13.6	57,340	39,930	9.5	17,405	8.5	57,335	27,935	9.3	29,405	9.0	57,340
	South East	34,145	6.4	6375	6.6	40,520	24,440	5.8	16,080	7.9	40,520	14,720	4.9	25,800	7.9	40,520
	South West	64,915	12.3	12,600	13.1	77,515	48,005	11.4	29,515	14.5	77,515	32,775	10.9	44,735	13.8	77,510
	West Midlands	22,205	4.2	4905	5.1	27,110	20,020	4.8	7085	3.5	27,105	15,145	5.0	11,960	3.7	27,105
	Yorkshire and The Humber	88,205	16.7	13,185	13.7	101,390	72,590	17.2	28,810	14.1	101,400	52,795	17.6	48,595	14.9	101,390
Ethnicity	Asian or Asian British	57,370	10.8	10,650	11.1	68,020	48,055	11.4	19,965	9.8	68,020	34,990	11.6	33,030	10.1	68,020
	Black or Black British	16,240	3.1	3110	3.2	19,350	13,470	3.2	5880	2.9	19,350	10,070	3.3	9280	2.9	19,350
	Chinese or Other Ethnic Groups	11,990	2.3	2165	2.2	14,155	9950	2.4	4205	2.1	14,155	7330	2.4	6825	2.1	14,155
	Mixed	10,670	2.0	1975	2.1	12,645	8765	2.1	3880	1.9	12,645	6525	2.2	6120	1.9	12,645
	White	431,720	81.5	78,060	81.1	509,785	339,905	80.6	169,880	83.1	509,785	240,575	80.0	269,210	82.7	509,785
	Missing	1905	0.4	320	0.3	2225	1545	0.4	680	0.3	2225	1110	0.4	1115	0.3	2225
Index of Multiple Deprivation (IMD)	1 (most deprived)	131,485	24.8	22,215	23.1	153,700	111,505	26.4	42,275	20.7	153,780	81,280	27.0	72,405	22.2	153,690
	2	108,465	20.5	19,360	20.1	127,825	87,630	20.8	40,140	19.6	127,770	62,340	20.7	65,490	20.1	127,830
	3	101,195	19.1	19,060	19.8	120,255	78,850	18.7	41,425	20.3	120,275	55,980	18.6	64,260	19.7	120,240
	4	88,995	16.8	16,930	17.6	105,920	68,445	16.2	37,480	18.3	105,925	48,195	16.0	57,785	17.7	105,985
	5 (least deprived)	75,650	14.3	13,960	14.5	89,610	56,940	13.5	32,620	16.0	89,560	40,340	13.4	49,255	15.1	89,595
	Missing	24,110	4.5	4755	4.9	28,865	18,320	4.3	10,555	5.2	28,875	12,460	4.1	16,390	5.0	28,850
Charlson Comorbidity Index’s Group	Zero	457,460	86.3	82,450	85.6	539,910	364,440	86.4	175,440	85.8	539,880	259,685	86.4	280,180	86.1	539,865
	Low	67,265	12.7	12,610	13.1	79,875	53,130	12.6	26,755	13.1	79,885	37,885	12.6	42,020	12.9	79,905
	Medium	3510	0.7	650	0.7	4165	2755	0.7	1395	0.7	4150	1965	0.7	2175	0.7	4140
	High	1400	0.3	480	0.5	1880	1125	0.3	755	0.4	1880	885	0.3	1010	0.3	1895
	Very high	265	0.1	90	0.1	355	245	0.1	140	0.1	385	180	0.1	195	0.1	375

The most common delivery codes recorded were “Spontaneous vertex delivery”, “Single live birth” and “Delivery normal”, and the most common postnatal codes were “Maternal postnatal 6 week examination”, “Postnatal examination normal” and “Postnatal maternal examination”. For a summary of the ten most frequent codes, see Additional file 1: Tab.S2.

### Rate of postnatal examinations

**Fig. 1:**
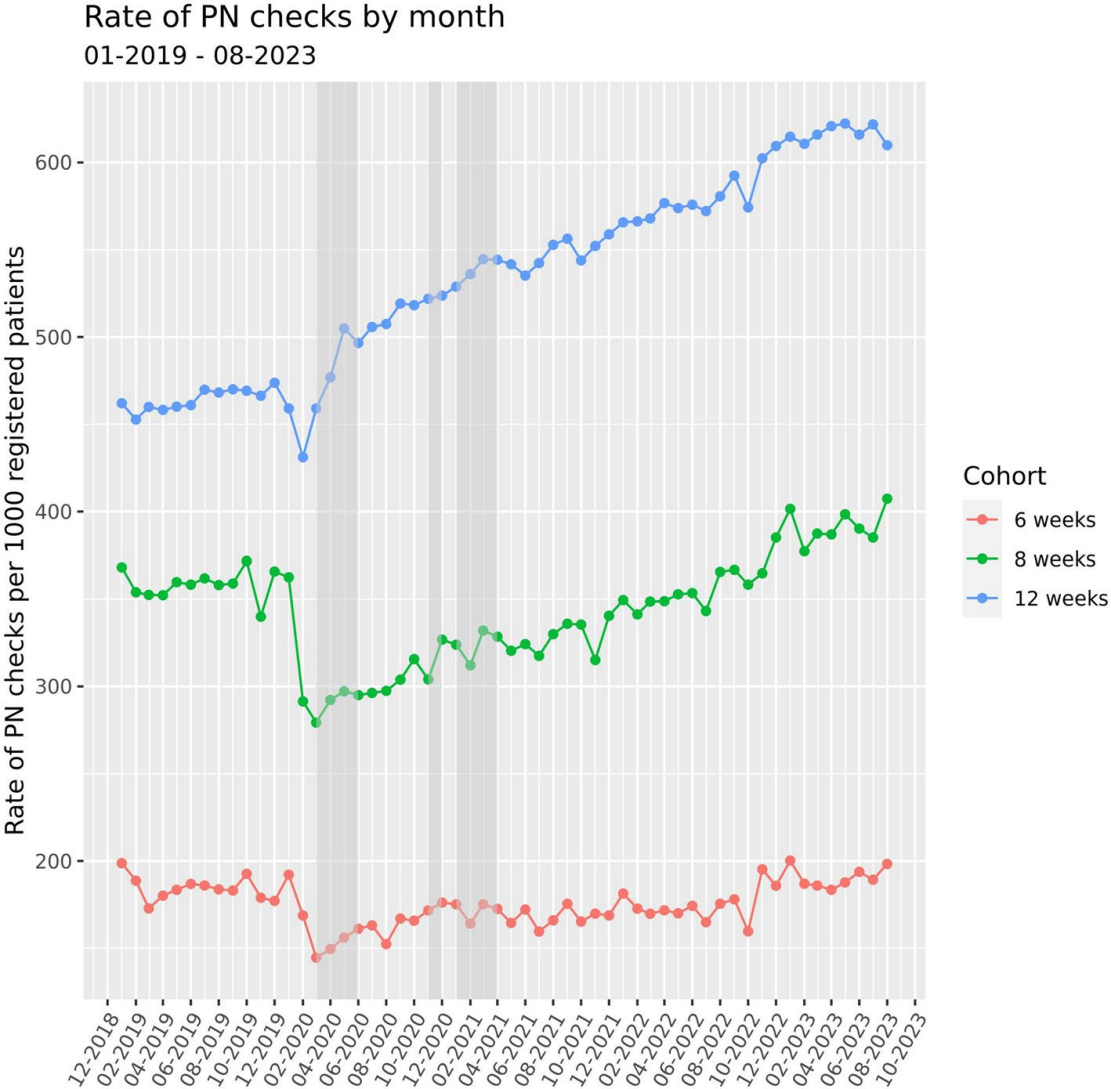
The rate of postnatal (PN) examinations per 1000 unique patients reduced during the pandemic. Rate was calculated for each calendar month using the delivery date as the reference point assessing whether PN checks occurred within 6, 8 or 12 weeks following a delivery code. The proportion of PN checks within the defined follow-up period was estimated relative to the total number of deliveries in that index month. Due to this calculation method, the initial reduction observed in PN examinations during the national lockdown periods (indicated by grey-shaded bars), appear as a reduction in the months prior to the lockdowns

The rate of postnatal examinations reduced at the start of the COVID-19 pandemic. For examinations within 6 weeks, the greatest reduction was from 192.1 to 144 per 1000 deliveries, equating to a 24.7% drop between January and March 2020. For examinations within 8 weeks, the greatest fall was from 368.2 to 279.3 per 1000 deliveries, equating to a 24.1% drop between January and March 2020. Rates for 6 and 8 weeks remained low but fluctuated throughout the national lockdown periods and eventually recovered to pre-pandemic rates around November 2022 (Fig. 1), suggesting 200 and 400 per 1000 new mothers received postnatal examinations within the recommended 6-8 weeks.

**Fig. 2:**
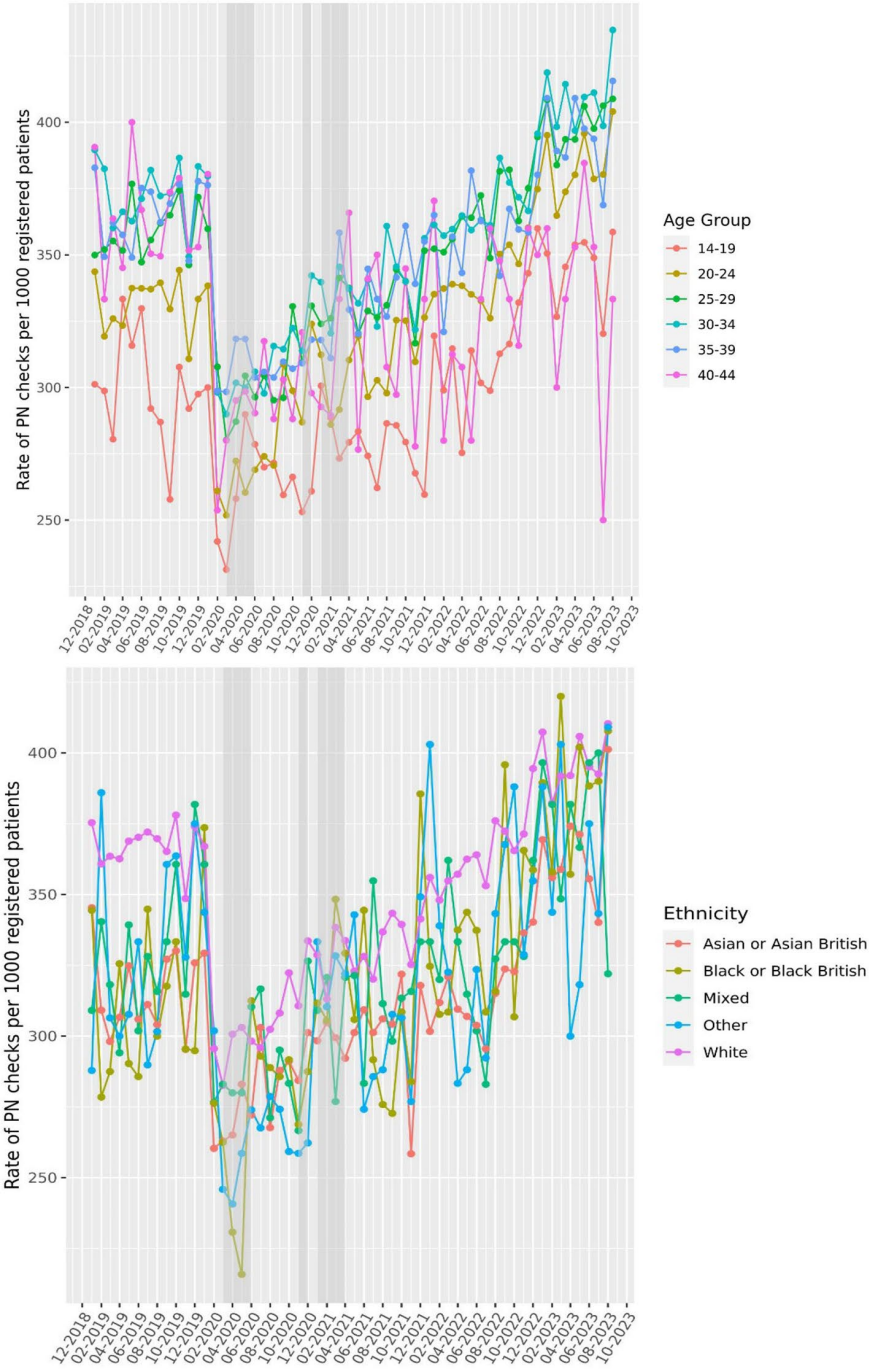
The rate of postnatal (PN) examinations per 1000 unique patients stratified by age or ethnic group. Rate of examinations was calculated for each calendar month, stratified by age or ethnicity, using the delivery date as the reference point assessing whether PN checks occurred within 6, 8 or 12 weeks following a delivery code. The proportion of PN checks within the defined follow-up period was estimated relative to the total number of deliveries in that index month. Due to this calculation method, the initial reduction observed in PN examinations during the national lockdown periods (indicated by grey-shaded bars), appear as a reduction in the months prior to the lockdowns

**Fig. 3:**
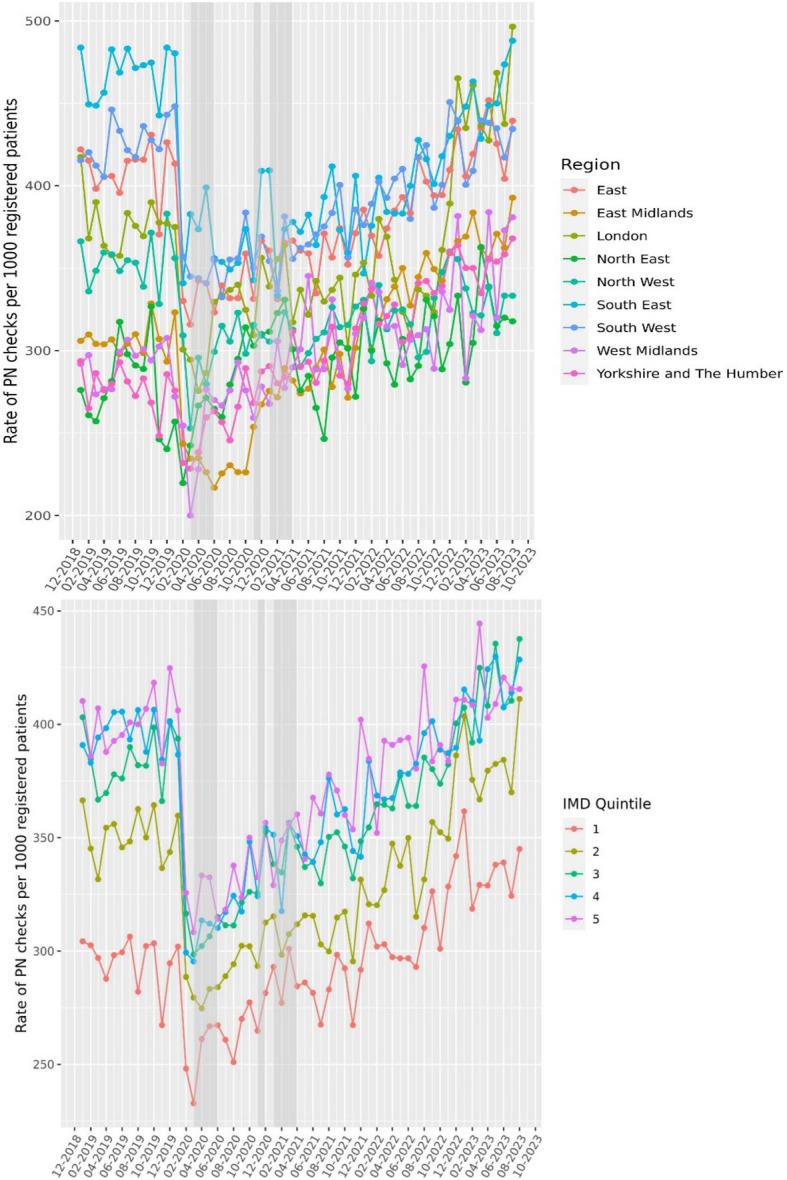
The rate of postnatal (PN) examinations per 1000 unique patients stratified by region or index of multiple deprivation (IMD) quintile. Rate of examinations was calculated for each calendar month, stratified by region or IMD, using the delivery date as the reference point assessing whether PN checks occurred within 6, 8 or 12 weeks following a delivery code. The proportion of PN checks within the defined follow-up period was estimated relative to the total number of deliveries in that index month. Due to this calculation method, the initial reduction observed in PN examinations during the national lockdown periods (indicated by grey-shaded bars), appear as a reduction in the months prior to the lockdowns

Pre-pandemic rates were higher for postnatal checks within 12 weeks follow-up (473.7 per 1000 in December 2019) and reduced initially but recovered quickly. Rates within 12 weeks continued to rise, reaching and surpassing pre-pandemic levels to 622.3 per 1000 in May 2023 (an increase of 31.4%). Rates also varied by maternal demographics. By age, rates were lower for younger and older groups (< 24 and ≥ 40 years). Rates were lower and remain lower for women residing in the North East, North West, West Midlands and Yorkshire regions of England. Women from ethnic minorities also had fewer appointments over time with a pronounced reduction during the national lockdown periods. Similarly, rates were lower in women from the bottom two deprivation quintiles across the entire study period (Figs. 2 and 3).

### The impact of the pandemic on rates

Interrupted time series showed reduced incident rate ratios (IRRs) at the start of the pandemic of 0.87 (95% confidence interval (CI): 0.83–0.91) for 6 weeks and 0.84 (0.81–0.87) for 8 weeks, with a small increase to IRRs for 12 weeks (1.06; CI 1.04–1.08) (Table 2). Adjusting for short-term autocorrelation using Newey-West robust standard errors had minimal impact on point estimates: the immediate drop in postnatal examinations at pandemic onset remained unchanged (IRR of 0.87 and 0.84 for 6 and 8 weeks, respectively), although the 95% CI widened slightly, reflecting a more conservative estimate of uncertainty (Additional file 1: Tab.S3). The greatest reduction in IRRs was observed for age category 25–29 years (IRR 0.83 (0.79–0.86)) and for ethnicity classified as “Other” (IRR 0.77 (0.68–0.87)). IRRs for deprivation varied by quintiles from 0.81 (0.78–0.85) for quintile 3 to 0.91 (0.86–0.95) in the most deprived quintile 1. The counterfactual, reflecting the expected trend if no interruption had occurred, demonstrated a steady or continued downward trajectory in the rate of postnatal examinations overall (Additional file 1: Fig. S1) and by demographic sub-group.

**Table 2:** Incident rate ratios (IRR) for the rate of postnatal examinations. IRRs were estimated comparing pre-pandemic rates with the first national lock-down period modelled as an interruption in an interrupted time series analysis for COVID-19. IRRs by demographic are displayed for the 8-week follow-up cohort

	IRR	95% confidence interval	
6-weeks	0.87	0.83	0.91
8-weeks	0.84	0.81	0.87
12-weeks	1.06	1.04	1.08
**Age category (year)**			
14–19	0.89	0.82	0.98
20–24	0.84	0.80	0.89
25–29	0.83	0.79	0.86
30–34	0.84	0.80	0.88
35–39	0.84	0.80	0.89
40–44	0.86	0.77	0.95
**Ethnicity**			
White	0.84	0.80	0.87
Mixed	0.81	0.72	0.92
Asian or Asian British	0.88	0.83	0.94
Black or Black British	0.83	0.74	0.92
Other	0.77	0.68	0.87
**Deprivation**			
Most deprived—1	0.91	0.86	0.95
2	0.82	0.78	0.86
3	0.81	0.78	0.85
4	0.83	0.79	0.87
Least deprived—5	0.82	0.78	0.86
**Region**			
East	0.83	0.79	0.87
East Midlands	0.76	0.71	0.81
London	0.84	0.76	0.92
North East	1.05	0.96	1.16
North West	0.85	0.80	0.90
South East	0.79	0.74	0.85
South West	0.80	0.76	0.84
West Midlands	0.90	0.82	0.98
Yorkshire and The Humber	0.96	0.91	1.01

### The association of characteristics and no postnatal examination

The odds of no postnatal examination in the 8 weeks after birth were greater for women of ethnic minorities compared to White women (Table 3). For Chinese or Other, the odds ratio was 1.16 (95% confidence interval: 1.11–1.21), Asian 1.11 (1.09–1.14), Black 1.08 (1.03–1.12), Mixed 1.08 (1.03–1.14). The odds of no postnatal examination were also greater for the more deprived quintiles compared to the least deprived. The odds of not having a timely postnatal examination were 34% higher for the most deprived quintile compared to the least deprived, and 21% higher for the second most deprived quintile.

**Table 3:** Modelling the association of patient characteristics for no postnatal examination within 6-, 8- or 12-week follow-up. Data presented as odds ratio (OR) and 95% confidence intervals (CI)

	6-weeks				8-weeks^*^				12-weeks			
	OR	95% CI		*P* value	OR	95% CI		*P* value	OR	95% CI		*P* value
(Intercept)	4.06	3.78	4.35		1.72	1.62	1.83	0.00	0.88	0.84	0.93	
Age	0.998	0.996	0.999	0.002	0.999	0.998	1.000	0.228	1.010	1.009	1.011	< 0.001
BMI	0.996	0.994	0.997	< 0.001	0.996	0.995	0.997	< 0.001	0.996	0.995	0.997	< 0.001
Ethnicity												
Asian or Asian British	1.03	1.00	1.06	0.03	1.11	1.09	1.14	< 0.001	1.02	1.00	1.04	0.06
Black or Black British	1.01	0.97	1.06	0.60	1.08	1.03	1.12	< 0.001	1.06	1.02	1.10	< 0.001
Chinese or Other Ethnic Groups	1.07	1.01	1.13	0.01	1.16	1.11	1.21	< 0.001	1.09	1.05	1.14	< 0.001
Mixed	1.03	0.97	1.10	0.28	1.08	1.03	1.14	< 0.001	1.13	1.08	1.18	< 0.001
Deprivation quintile								< 0.001				< 0.001
1	1.08	1.05	1.11	< 0.001	1.34	1.31	1.37	< 0.001	1.24	1.22	1.27	< 0.001
2	1.06	1.03	1.09	< 0.001	1.21	1.18	1.24	< 0.001	1.11	1.09	1.13	< 0.001
3	1.01	0.98	1.04	0.45	1.10	1.07	1.12	< 0.001	1.06	1.04	1.08	< 0.001
4	0.97	0.95	1.00	0.06	1.04	1.02	1.07	< 0.001	1.02	1.00	1.04	0.11
Region												
East	1.56	1.51	1.62	< 0.001	0.94	0.91	0.97	< 0.001	0.61	0.59	0.63	< 0.001
East Midlands	1.76	1.70	1.83	< 0.001	1.32	1.28	1.37	< 0.001	1.04	1.01	1.07	0.01
North East	1.77	1.69	1.87	< 0.001	1.39	1.33	1.45	< 0.001	0.99	0.96	1.03	0.67
North West	1.52	1.46	1.58	< 0.001	1.19	1.15	1.24	< 0.001	0.80	0.77	0.83	< 0.001
South East	1.51	1.44	1.58	< 0.001	0.82	0.79	0.85	< 0.001	0.49	0.48	0.51	< 0.001
South West	1.47	1.42	1.53	< 0.001	0.89	0.86	0.92	< 0.001	0.65	0.63	0.67	< 0.001
West Midlands	1.23	1.17	1.29	< 0.001	1.33	1.27	1.39	< 0.001	1.01	0.97	1.05	0.62
Yorkshire and The Humber	1.85	1.78	1.92	< 0.001	1.27	1.23	1.32	< 0.001	0.90	0.88	0.93	< 0.001
Charlson comorbidity Index Score > 0	0.93	0.90	0.97	< 0.001	1.01	0.98	1.04	0.52	1.09	1.06	1.12	< 0.001
Covid time					1.07	1.05	1.08	< 0.001				

When patients had more than one delivery code in the study period one was selected at random to model

Reference categories for ethnicity: White, Deprivation quintile: 5 least deprived, region: London, Charlson comorbidity index score of zero

^*^The model for 8 weeks follow-up was also adjusted for covid time where a value of one indicated a delivery from March 2020, and the reference category zero indicated before March 2020

The association between no examination and region varied substantially; for example, the odds for no postnatal examination in the recommended 6 weeks was 1.76 (1.70–1.83) for the East Midlands compared to London, but just 1.04 (1.01–1.07) within 12 weeks. Similarly, the odds were 0.49 (0.48–0.51) in 12 weeks follow-up in South East, but 1.51 in 6 weeks. This suggests most regions can accommodate examinations within a longer follow-up period, but some regions record more examinations in the recommended time than others. Furthermore, older women and women with a comorbidity were more likely to receive a timely examination compared to younger, healthier individuals (Table 3). Comparing associations of risk before and after the pandemic (2019 v 2022), age and BMI remain unchanged; however, the risk in some regions compared to London has improved. The odds of no examination for deprived socioeconomic groups remain and increased for a postnatal examination within 6-8 weeks for the North West and South East, those with existing comorbidity and Asian ethnicity (Additional file 1: Tab.S4).

## Discussion

This study observed a substantial reduction in the rate of postnatal examinations in the first lockdown period of the COVID-19 pandemic in England. Rates fluctuated before gradually returning to pre-pandemic levels. However, the recovery of service delivery varied, showing that fewer patients received a postnatal examination in the recommended 6-8 weeks compared to 12 weeks follow-up throughout the pandemic and beyond. Furthermore, there were persistent regional, socioeconomical and ethnic disparities in accessing the routine postnatal check. These factors also remain significantly associated with the risk of no postnatal examination.

### Reduction in postnatal care during the pandemic

The significant reduction observed in this study at the onset of the COVID-19 pandemic corresponds with the imposed lockdown and widespread restrictions in place across in England. Similar findings have been observed globally, including the initial changes to healthcare delivery and public behaviours. Routine healthcare was deprioritised as resources were redirected to containing the spread and managing the clinical impact of COVID-19 infections. Numerous studies have demonstrated similar findings in access and delivery of routine healthcare during this period [20–22].

Despite the interruption caused by the initial lockdown periods of the pandemic, the study showed a subsequent upward trajectory as healthcare services recovered. The upward trajectory demonstrated an improvement in rates of examinations within 6 or 8 weeks similar to 2019 by the end of 2022; however, recovery for examinations within 12 weeks was much quicker. This upward trend may not have been solely due to post-lockdown recovery but could also be attributed to other factors, such as a policy change implemented prior to the start of the pandemic. The general practitioner (GP) contract was updated in February 2020 to include a standardised 6-to-8 week postnatal check for new mothers, with the addition of £12 million in funding to support the change [4]. This change aimed to ensure that the postnatal check was offered as an essential service to all mothers as a separate appointment in addition to the routine baby check appointments during this period. The pandemic likely stalled the effects of the initial change in contract by limiting access to care in the first few months. At the same time, births in England and Wales fell from ~657,000 in 2019 to ~614,000 in 2020, with a modest recovery in 2021 (~625,000) before declining again (~605,000 in 2022; 591,000 in 2023; est. ~568,000 in 2024) [23]. While the total number of live births declined, the proportion of deliveries receiving a postnatal examination initially fell and later recovered, likely reflecting both the GP contractual changes and reduced demand. Nevertheless, disparities in access and delays in examinations remain, highlighting persistent inequities in care or patients’ ability to attend appointments.

### Regional, socioeconomic and ethnic disparities

The analysis showed significant variation in postnatal care across regions, deprivation quintiles and between ethnic groups. Whilst these changes reflected the pandemic’s impact on routine antenatal care, factors that increase the likelihood of having late or no postnatal examination overlap with many risk factors for adverse outcomes of COVID-19 infections, such as ethnic minority groups and deprivation, meaning that the consequences of these changes may not have been evenly distributed. For example, ethnic minority groups were shown to have an increased risk of suffering more serious COVID-19 infections and outcomes [24]. As a result, the initial reduction in postnatal examinations for these groups may have been related to increased concern around contracting COVID-19 and were more likely to avoid appointments. However, comparing the risk before and after the pandemic recovery, in 2022, the risk of missing appointments remained higher for ethnic minority groups compared to White women, suggesting there are other factors contributing to the observed differences.

The Incidence Rate Ratios (IRRs) were not as pronounced for some groups, such as the most deprived quintile. The small reduction in IRR is likely because this group already exhibited the lowest rate of postnatal examinations before the onset of the pandemic. In contrast, more affluent groups experienced a substantial reduction in postnatal care appointments during the onset of the pandemic. This suggests that the abrupt change in healthcare delivery disproportionately affected those who previously had better access to care, whilst those with existing poor access or uptake were less affected. The eventual recovery in the rate of postnatal examinations did not eliminate the pre-existing disparities in postnatal care access or uptake, which were also significant for women from ethnic minority groups and younger women.

These findings align with longstanding research showing socioeconomic and ethnic disparities in healthcare access [25] and uptake [26], especially for maternal and perinatal care and health outcomes [27]. The MBRRACE-UK reports into maternal deaths and morbidity show Asian and Black women are two and four times more likely to die during pregnancy or up to 6 weeks after childbirth [28]. This association was also evident for infant mortality in ethnic minorities and deprived regions [29]. Similarly, a recent study highlighted in the introduction found that postnatal 6-to-8 week baby check appointments were less frequent among ethnic minority groups in the UK, even after accounting for other risk factors such as region [13]. It was also shown that infants were less likely to have a check if mothers also had no record of a postnatal maternal examination. This raises the question of whether these patient groups are less likely to be invited, or more probably, less likely to take up the invitation, suggesting potential barriers related to access, awareness and culturally sensitive follow-up care in the community, particularly if information about postnatal services was communicated in ways that were not inclusive.

The current study also highlighted regional disparities in postnatal care, with areas such as the North East, East and West Midlands and Yorkshire and The Humber having particularly lower rates of postnatal examinations. Access to care may be worse for ethnic minority groups in these regions, especially if existing challenges, such as deprivation, health illiteracy, language barriers, digital exclusion and culturally insensitive communication, are not addressed [30–32]. Efforts to promote equitable care postnatally should prioritise addressing these barriers and ensuring that care delivery is culturally sensitive and accessible to all.

The impact of delayed or no postnatal examinations on immediate and long-term maternal and infant health may well be exacerbated in marginalised communities, clustered in regions of the UK. There is evidence to support inequalities in outcomes of pregnancy and childbirth in these groups, such as an increased risk of small for gestational age babies, with a higher incidence of low Apgar scores (an assessment of a newborn's health performed shortly after birth) and more neonatal admissions [33, 34].

### Policy implications and recommendations

While some recovery has been observed following the pandemic's initial phase, disparities in access or uptake remain. These findings demonstrate the urgent need for healthcare systems to prioritise equitable access to, and uptake of, postnatal care, particularly for socioeconomically disadvantaged and ethnic minority groups. This is critical due to the higher rates of maternal and infant mortality in these populations. This is particularly challenging when the number of GPs per patient also varies by local area, with 15% more patients to GPs in more deprived areas, equating to 370 more patients per single GP [35]. Further support to primary care services or expansion of postnatal follow-up within women’s health hubs is urgently needed to sustain postnatal follow-up, especially if the service will remain within an already overburdened primary care system [36, 37].

The observed disparity is highly likely to have significant implications for maternal and infant health outcomes, as maternal mortality is highest in the first 42 days postpartum [38]. Timely postnatal examinations are critical for identifying and managing complications. For example, achieving blood pressure (BP) control within 6 weeks is associated with a persistently lower BP at 6 months [39] and at 4 years, with an estimated 30% reduction in subsequent cardiovascular risk [40]. Echocardiographic improvements are also evident by 6 weeks but show little change from 6 weeks to 6 months [41], suggesting early control may limit vascular remodelling. Similarly, postpartum diabetes screening at 4–12 weeks enables earlier detection of dysglycaemia and timely intervention. Given that cardiometabolic conditions like hypertensive disorders of pregnancy and gestational diabetes are both common and disproportionately affect minoritised populations [42–44], equitable and timely postnatal assessment is essential to reduce long-term cardiometabolic risk. Further work is needed to understand if these disparities stem from certain patient groups not being offered appointments, not being offered them in a timely manner, or not taking up these appointments when offered—or a combination of these factors. Further understanding will help guide interventions to improve appointment offerings, as well as uptake and access for socioeconomically disadvantaged and ethnic minority groups.

Efforts to improve access and uptake of postnatal care should focus on immediate interventions for at-risk populations, alongside long-term strategies and early postnatal preventative action to address systemic health inequalities. However, financial incentives alone may not be sufficient; tailored interventions are also needed to address the unique needs of disadvantaged populations by targeting areas with the highest health inequalities [31]. This includes improving outreach to communities with low health literacy, language barriers and limited digital access [45]. Such change in the postnatal period likely needs effective collaboration and communication between maternity and primary care services.

Furthermore, improving health between pregnancies is an opportunity to prevent poor outcomes in subsequent pregnancies and in women’s later life, but requires timely interventions to address modifiable risk factors. This includes prioritising care for individuals at immediate risk, as well as those with long-term risk of developing comorbidities. Enhanced processes are needed to optimise care delivery and reduce the burden on the healthcare system, ensuring that high-risk groups receive targeted, effective interventions [31].

### Strengths and limitations

These observations are based on comprehensive data from over 2500 primary care practices covering 43% of the English population [46]. This study provides valuable insights into postnatal care trends before, during and after the COVID-19 pandemic. However, it is important to note some limitations. The study relied on coded entries in the patient electronic health records to identify postnatal healthcare interactions, which may have led to an underestimation of interactions if they were not coded, documented in free text or coincided with the baby check appointment. Furthermore, the follow-up period (within 6, 8 and 12 weeks of a delivery code) may have missed checks that occurred after the follow-up. Conversely, the inclusion of all recorded postnatal examinations, including those in the community, may have led to a slight overestimation. Furthermore, in sensitivity analyses, we accounted for short-term autocorrelation using Newey-West robust standard errors, which had minimal impact on point estimates, though confidence intervals widened slightly. We did not adjust for seasonality; while England shows modest birth seasonality with a slight late-summer/early-autumn peak, the abrupt pandemic-related drop is unlikely to be explained by either factor. Our regression analyses used complete-case data. Missingness was very low for variables such as ethnicity (< 0.4%) and IMD (< 5%) and slightly higher for BMI (< 18.5%). While multiple imputation could not be performed due to the large size of the monthly extracts, this is unlikely to substantially affect the results. Complete-case analysis may introduce bias if missingness is related to maternal characteristics or service use, representing a potential limitation of the study. Despite this limitation, the study offers a comprehensive overview of postnatal care during a period of significant disruption and the status of healthcare provisions. Further research should explore the direct impact on long-term health for women who experience delayed or missed postnatal healthcare examinations across different patient populations to fully understand the broader health consequences.

## Conclusion

The COVID-19 pandemic significantly disrupted postnatal care provision, with the greatest reductions observed during the initial phase. While there has been a recovery in service provision, disparities in access or uptake of care remain, particularly among socioeconomically disadvantaged and ethnic minority groups. Addressing these disparities will require a combination of policy changes, financial incentives and targeted, culturally appropriate interventions to promote equitable access to care for all mothers and infants.

## Supplementary Information

Additional File 1: Additional Tables and Figures. Table S1: Charlson comorbidity characteristics of the population. Table S2: Ten most frequent birth and postnatal codes within the study period. Table S3: Incident rate ratios (IRR) for the rate of postnatal checks with autocorrelation adjustment. Figures S1: Interrupted Time Series analysis of the rate of postnatal examinations 6-, 8-, or 12-weeks follow-up cohorts with counterfactual. Table S4: Incident rate ratios (IRR) for the rate of postnatal examinations within 8-weeks, 2019 compared to 2020.

## Data Availability

Access to the underlying identifiable and potentially re-identifiable pseudonymised electronic health record data is tightly governed by various legislative and regulatory frameworks, and restricted by best practice. The data in the NHS England OpenSAFELY COVID-19 service is drawn from General Practice data across England where TPP is the data processor. TPP developers initiate an automated process to create pseudonymised records in the core OpenSAFELY database, which are copies of key structured data tables in the identifiable records. These pseudonymised records are linked onto key external data resources that have also been pseudonymised via SHA-512 one-way hashing of NHS numbers using a shared salt. University of Oxford, Bennett Institute for Applied Data Science developers and PIs, who hold contracts with NHS England, have access to the OpenSAFELY pseudonymised data tables to develop the OpenSAFELY tools. These tools in turn enable researchers with OpenSAFELY data access agreements to write and execute code for data management and data analysis without direct access to the underlying raw pseudonymised patient data, and to review the outputs of this code. All code for the full data management pipeline — from raw data to completed results for this analysis — and for the OpenSAFELY platform as a whole is available for review at github.com/OpenSAFELY.

## Abbreviations

BMI: Body mass index
CI: Confidence interval
COPD: Chronic obstructive pulmonary disease
COVID-19: Coronavirus disease of 2019
EHR: Electronic health records
GP: General practitioner
IMD: Index of multiple deprivation
IRR: Incidence rate ratio
ITS: Interrupted time series
LSOA: Lower Super Output Area
MBRRACE: Mothers and Babies: Reducing Risk through Audits and Confidential Enquiries
NHS: National Health Service
NICE: National Institute for Health and Care Excellence
UK: United Kingdom

## References

[1] National Institute for Health and Care Excellence (NICE). Postnatal care: Recommendations, Assessment and care of the woman 2021. Available from: https://www.nice.org.uk/guidance/ng194/chapter/recommendations#assessment-and-care-of-the-woman.

[2] NHS England. GP six to eight week maternal postnatal consultation – what good looks like guidance 2023. Available from: https://www.england.nhs.uk/long-read/gp-six-to-eight-week-maternal-postnatal-consultation-what-good-looks-like-guidance/.

[3] NHS England. Overview | Postnatal care | Guidance | NICE 2021. Available from: https://www.nice.org.uk/guidance/ng194.

[4] NHS England. NHS England » Investment and Evolution: Update to the GP contract agreement 2020/21 – 2023/24 2020. Available from: https://www.england.nhs.uk/publication/investment-and-evolution-update-to-the-gp-contract-agreement-20-21-23-24/.

[5] Jakes AD, Oakeshott P, Bick D. The maternal six week postnatal check. BMJ. 2019;367:l6482. 31791954 10.1136/bmj.l6482

[6] Li Y, Kurinczuk JJ, Gale C, Siassakos D, Carson C. Evidence of disparities in the provision of the maternal postpartum 6-week check in primary care in England, 2015–2018: an observational study using the Clinical Practice Research Datalink (CPRD). J Epidemiol Community Health. 2022;76(3):239–46. 34503988 10.1136/jech-2021-216640 PMC8862061

[7] UK Health Security Agency. Coronavirus (COVID-19): guidance 2020. Available from: https://www.gov.uk/government/collections/coronavirus-covid-19-list-of-guidance.

[8] Goyal M, Singh P, Singh K, Shekhar S, Agrawal N, Misra S. The effect of the COVID-19 pandemic on maternal health due to delay in seeking health care: experience from a tertiary center. Int J Gynaecol Obstet. 2021;152(2):231–5. 33128794 10.1002/ijgo.13457 PMC9087665

[9] Fonseca M, MacKenna B, Mehrkar A, Open SC, Walters CE, Hickman G, et al. The Use of Online Consultation Systems or Remote Consulting in England Characterized Through the Primary Care Health Records of 53 Million People in the OpenSAFELY Platform: Retrospective Cohort Study. JMIR Public Health Surveill. 2024;10:e46485. 39292500 10.2196/46485 PMC11447420

[10] Majeed A, Maile EJ, Bindman AB. The primary care response to COVID-19 in England’s National Health Service. J R Soc Med. 2020;113(6):208–10. 32521196 10.1177/0141076820931452 PMC7439588

[11] Townsend R, Chmielewska B, Barratt I, Kalafat E, van der Meulen J, Gurol-Urganci I, et al. Global changes in maternity care provision during the COVID-19 pandemic: a systematic review and meta-analysis. eClin Med. 2021;37:100947. 10.1016/j.eclinm.2021.100947 PMC8233134 34195576

[12] Knight M, Bunch K, Vousden N, Morris E, Simpson N, Gale C, et al. Characteristics and outcomes of pregnant women admitted to hospital with confirmed SARS-CoV-2 infection in UK: national population based cohort study. BMJ. 2020;369:m2107. 32513659 10.1136/bmj.m2107 PMC7277610

[13] Zhang CX, Quigley MA, Bankhead C, Kwok CH, Parekh N, Carson C. Ethnic inequities in 6–8 week baby check coverage in England 2006–2021: a cohort study using the clinical practice research datalink. Br J Gen Pract. 2024. 10.3399/BJGP.2023.0593. 38621807 10.3399/BJGP.2023.0593 PMC11289950

[14] Nab L, Schaffer AL, Hulme W, DeVito NJ, Dillingham I, Wiedemann M, et al. OpenSAFELY: a platform for analysing electronic health records designed for reproducible research. Pharmacoepidemiol Drug Saf. 2024;33(6):e5815. 38783412 10.1002/pds.5815 PMC7616137

[15] What is the dm+d? The NHS Dictionary of Medicines and Devices.

[16] Minassian C, Williams R, Meeraus WH, Smeeth L, Campbell OMR, Thomas SL. Methods to generate and validate a pregnancy register in the UK Clinical Practice Research Datalink primary care database. Pharmacoepidemiol Drug Saf. 2019;28(7):923–33. 31197928 10.1002/pds.4811 PMC6618019

[17] NHS England. Home - Technology reference update distribution -TRUD. Available from: https://isd.digital.nhs.uk/trud/users/guest/filters/0/home.

[18] Charlson ME, Pompei P, Ales KL, MacKenzie CR. A new method of classifying prognostic comorbidity in longitudinal studies: development and validation. J Chronic Dis. 1987;40(5):373–83. 3558716 10.1016/0021-9681(87)90171-8

[19] Bernal JL, Cummins S, Gasparrini A. Interrupted time series regression for the evaluation of public health interventions: a tutorial. Int J Epidemiol. 2017;46(1):348–55. 27283160 10.1093/ije/dyw098 PMC5407170

[20] Filip R, Gheorghita Puscaselu R, Anchidin-Norocel L, Dimian M, Savage WK. Global challenges to public health care systems during the COVID-19 pandemic: a review of pandemic measures and problems. J Pers Med. 2022. 10.3390/jpm12081295. 36013244 10.3390/jpm12081295 PMC9409667

[21] Pujolar G, Oliver-Angles A, Vargas I, Vazquez ML. Changes in access to health services during the COVID-19 pandemic: a scoping review. Int J Environ Res Public Health. 2022. 10.3390/ijerph19031749. 35162772 10.3390/ijerph19031749 PMC8834942

[22] Tu K, Lapadula MC, Apajee J, Bonilla AO, Baste V, Cuba-Fuentes MS, et al. Changes in reasons for visits to primary care after the start of the COVID-19 pandemic: an international comparative study by the International Consortium of Primary Care Big Data Researchers (INTRePID). PLOS Glob Public Health. 2024;4(8):e0003406. 39173045 10.1371/journal.pgph.0003406 PMC11341054

[23] Office for National Statistics. Live births. Available from: https://www.ons.gov.uk/peoplepopulationandcommunity/birthsdeathsandmarriages/livebirths.

[24] Siddiq S, Ahmed S, Akram I. Clinical outcomes following COVID-19 infection in ethnic minority groups in the UK: a systematic review and meta-analysis. Public Health. 2023;222:205–14. 35970621 10.1016/j.puhe.2022.05.019 PMC9181265

[25] Wheeler SM, Bryant AS. Racial and ethnic disparities in health and health care. Obstet Gynecol Clin North Am. 2017;44(1):1–11. 28160887 10.1016/j.ogc.2016.10.001

[26] Molokhia M, Ayis DS, Karamanos A, L’Esperance DV, Yousif S, Durbaba S, et al. What factors influence differential uptake of NHS health checks, diabetes and hypertension reviews among women in ethnically diverse South London? Cross-sectional analysis of 63,000 primary care records. eClinicalMedicine. 2022;49:101471. 35747176 10.1016/j.eclinm.2022.101471 PMC9156982

[27] Howell EA, Zeitlin J. Quality of care and disparities in obstetrics. Obstet Gynecol Clin North Am. 2017;44(1):13–25. 28160890 10.1016/j.ogc.2016.10.002 PMC5300700

[28] MBRRACE-UK;. Perinatal Mortality Surveillance: UK perinatal deaths of babies born in 2021 2023. Available from: https://www.npeu.ox.ac.uk/mbrrace-uk/reports/perinatal-mortality-surveillance.

[29] Office for National Statistics. Births and infant mortality by ethnicity in England and Wales: 2007 to 2019. 2021.

[30] Forster AS, Rockliffe L, Chorley AJ, Marlow LAV, Bedford H, Smith SG, et al. Ethnicity-specific factors influencing childhood immunisation decisions among Black and Asian minority ethnic groups in the UK: a systematic review of qualitative research. J Epidemiol Community Health. 2017;71(6):544–9. 27531844 10.1136/jech-2016-207366 PMC5484038

[31] O’Connor S, Tilston G, Jones O, Sharma A, Ormesher L, Quinn B, et al. Acceptability of data linkage to identify women at risk of postnatal complication for the development of digital risk prediction tools and interventions to better optimise postnatal care, a qualitative descriptive study design. BMC Med. 2024;22(1):276. 38956666 10.1186/s12916-024-03489-7 PMC11220952

[32] Gardiner T, Abraham S, Clymer O, Rao M, Gnani S. Racial and ethnic health disparities in healthcare settings. BMJ. 2021;372:n605. 33685939 10.1136/bmj.n605

[33] National Maternity & Perinatal Audit. Ethnic and socio-economic inequalities in NHS maternity and perinatal care for women and their babies 2021.

[34] Jardine J, Walker K, Gurol-Urganci I, Webster K, Muller P, Hawdon J, et al. Adverse pregnancy outcomes attributable to socioeconomic and ethnic inequalities in England: a national cohort study. Lancet. 2021;398(10314):1905–12. 34735797 10.1016/S0140-6736(21)01595-6

[35] The Health Foundation. A worrying cycle of pressure for GPs in deprived areas 2019. Available from: https://www.health.org.uk/news-and-comment/blogs/a-worrying-cycle-of-pressure-for-gps-in-deprived-areas.

[36] Macdonald C, Sharma S, Kallioinen M, Jewell D. Postnatal care: new NICE guideline for the “Cinderella service.” Br J Gen Pract. 2021;71(710):394–5. 34446406 10.3399/bjgp21X716825 PMC8378554

[37] Stewart S. Maternal postnatal care in general practice: steps forward. Br J Gen Pract. 2024;74(746):392–3. 39209714 10.3399/bjgp24X739161 PMC11349367

[38] Paladine HL, Blenning CE, Strangas Y. Postpartum care: an approach to the fourth trimester. Am Fam Physician. 2019;100(8):485–91. 31613576

[39] Cairns AE, Tucker KL, Leeson P, Mackillop LH, Santos M, Velardo C, et al. Self-management of postnatal hypertension: the SNAP-HT trial. Hypertension. 2018;72(2):425–32. 29967037 10.1161/HYPERTENSIONAHA.118.10911

[40] Kitt JA, Fox RL, Cairns AE, Mollison J, Burchert HH, Kenworthy Y, et al. Short-term postpartum blood pressure self-management and long-term blood pressure control: a randomized controlled trial. Hypertension. 2021;78(2):469–79. 34176288 10.1161/HYPERTENSIONAHA.120.17101 PMC8260340

[41] Ormesher L, Higson S, Luckie M, Roberts SA, Glossop H, Trafford A, et al. Postnatal cardiovascular morbidity following preterm pre-eclampsia: an observational study. Pregnancy Hypertens. 2022;30:68–81. 36029727 10.1016/j.preghy.2022.08.007

[42] Jiang L, Tang K, Magee LA, von Dadelszen P, Ekeroma A, Li X, et al. A global view of hypertensive disorders and diabetes mellitus during pregnancy. Nat Rev Endocrinol. 2022;18(12):760–75. 36109676 10.1038/s41574-022-00734-y PMC9483536

[43] Johnson JD, Louis JM. Does race or ethnicity play a role in the origin, pathophysiology, and outcomes of preeclampsia? An expert review of the literature. Am J Obstet Gynecol. 2022;226(2S):S876–85. 32717255 10.1016/j.ajog.2020.07.038

[44] Conti-Ramsden F, de Marvao A, Chappell LC. Pregnancy disorders and maternal consequences: ethnic disparities in hypertensive disorders of pregnancy. Reproduction. 2025. 10.1530/REP-25-0049. 40378303 10.1530/REP-25-0049 PMC12125630

[45] John JR, Curry G, Cunningham-Burley S. Exploring ethnic minority women’s experiences of maternity care during the SARS-CoV-2 pandemic: a qualitative study. BMJ Open. 2021;11(9):e050666. 34489290 10.1136/bmjopen-2021-050666 PMC8423508

[46] Andrews C, Schultze A, Curtis H, Hulme W, Tazare J, Evans S, et al. OpenSAFELY: Representativeness of electronic health record platform OpenSAFELY-TPP data compared to the population of England. Wellcome Open Res. 2022;7:191. 35966958 10.12688/wellcomeopenres.18010.1 PMC9346309

[47] NHS Digital (Now NHS England). The NHS England OpenSAFELY COVID-19 service - privacy notice 2023. Available from: https://digital.nhs.uk/coronavirus/coronavirus-covid-19-response-information-governance-hub/the-nhs-england-opensafely-covid-19-service-privacy-notice.

[48] NHS Digital (Now NHS England). Data Security and Protection Toolkit 2023. Available from: https://digital.nhs.uk/data-and-information/looking-after-information/data-security-and-information-governance/data-security-and-protection-toolkit

[49] NHS Digital (Now NHS England). ISB1523: Anonymisation Standard for Publishing Health and Social Care Data 2023. Available from: https://digital.nhs.uk/data-and-information/information-standards/information-standards-and-data-collections-including-extractions/publications-and-notifications/standards-and-collections/isb1523-anonymisation-standard-for-publishing-health-and-social-care-data

[50] Department of Health & Social Care. Coronavirus (COVID-19): notice under regulation 3(4) of the Health Service (Control of Patient Information) Regulations 2002 – general 2022. Available from: https://www.gov.uk/government/publications/coronavirus-covid-19-notification-of-data-controllers-to-share-information/coronavirus-covid-19-notice-under-regulation-34-of-the-health-service-control-of-patient-information-regulations-2002-general--2

[51] Secretary of State for Health and Social Care - UK Government. COVID-19 Public Health Directions 2020 2020. Available from: https://digital.nhs.uk/about-nhs-digital/corporate-information-and-documents/directions-and-data-provision-notices/secretary-of-state-directions/covid-19-public-health-directions-2020.

[52] NHS Health Research Authority. Confidentiality Advisory Group. Available from: https://www.hra.nhs.uk/about-us/committees-and-services/confidentiality-advisory-group/.

