# Supplementary material for: The impact of the COVID-19 pandemic on the rate of maternal postnatal healthcare examinations in England: an OpenSAFELY interrupted time series analysis providing evidence of disparity in care access
Source: BMC Med. 2025 Nov 10;23:626. doi: 10.1186/s12916-025-04436-w (PMC12599107; doi:10.1186/s12916-025-04436-w)
Supplement: Supplementary file 1 — Additional File 1: Additional Tables and Figures. Table S1: Charlson comorbidity characteristics of the population. Table S2: Ten most frequent birth and postnatal codes within the study period. Table S3: Incident rate ratios (IRR) for the rate of postnatal checks with autocorrelation adjustment. Figures S1: Interrupted Time Series analysis of the rate of postnatal examinations 6-, 8-, or 12-weeks follow-up cohorts with counterfactual. Table S4: Incident rate ratios (IRR) for the rate of postnatal examinations within 8-weeks, 2019 compared to 2020. [file 12916_2025_4436_MOESM1_ESM.docx]

# Additional Tables and Figures:

| Additional File 1- Tab.S1: Continued: detailed Charlson comorbidity characteristics of the population, stratified by follow-up cohort. | | | | | | | | | | | | | | | | | | | | | | |
| --- | --- | --- | --- | --- | --- | --- | --- | --- | --- | --- | --- | --- | --- | --- | --- | --- | --- | --- | --- | --- | --- | --- |
|  |  | 6-week cohort | | | | | |  | 8-week cohort | | | | | |  | 12-week cohort | | | | | | |
|  |  | Postnatal check | | | | |  |  | Postnatal check | | | | |  |  | Postnatal check | | | | |  |  |
|  |  | No | |  | Yes | | Total |  | No | |  | Yes | | Total |  | No | |  | Yes | |  | Total |
| Charlson | 0 | 457460 | **95.9** |  | 82450 | **95.6** | 539910 |  | 364440 | **95.9** |  | 175440 | **95.7** | 539880 |  | 259685 | **95.8** |  | 280180 | **95.9** |  | 539865 |
|  | 1 | 19635 | **4.1** |  | 3820 | **4.4** | 23455 |  | 15615 | **4.1** |  | 7835 | **4.3** | 23450 |  | 11430 | **4.2** |  | 12030 | **4.1** |  | 23455 |
| Any HBP | 0 | 518440 | **97.8** |  | 93740 | **97.4** | 612180 |  | 412625 | **97.8** |  | 199590 | **97.6** | 612215 |  | 293935 | **97.8** |  | 318235 | **97.7** |  | 612170 |
|  | 1 | 11465 | **2.2** |  | 2540 | **2.6** | 14005 |  | 9070 | **2.2** |  | 4900 | **2.4** | 13965 |  | 6665 | **2.2** |  | 7345 | **2.3** |  | 14010 |
| HBP in pregnancy | 0 | 524600 | **99.0** |  | 94945 | **98.6** | 619545 |  | 417525 | **99.0** |  | 202040 | **98.8** | 619565 |  | 297585 | **99.0** |  | 321955 | **98.9** |  | 619535 |
|  | 1 | 5300 | **1.0** |  | 1335 | **1.4** | 6635 |  | 4165 | **1.0** |  | 2450 | **1.2** | 6615 |  | 3020 | **1.0** |  | 3625 | **1.1** |  | 6645 |
| Cancer | 0 | 524265 | **98.9** |  | 95195 | **98.9** | 619460 |  | 417245 | **98.9** |  | 202215 | **98.9** | 619460 |  | 297465 | **99.0** |  | 321995 | **98.9** |  | 619460 |
|  | 2 | 5635 | **1.1** |  | 1085 | **1.1** | 6720 |  | 4445 | **1.1** |  | 2275 | **1.1** | 6720 |  | 3135 | **1.0** |  | 3585 | **1.1** |  | 6720 |
| Cardiovascular | 0 | 529310 | **99.9** |  | 96165 | **99.9** | 625480 |  | 421220 | **99.9** |  | 204265 | **99.9** | 625485 |  | 300255 | **99.9** |  | 325215 | **99.9** |  | 625470 |
|  | 1 | 590 | **0.1** |  | 115 | **0.1** | 700 |  | 470 | **0.1** |  | 225 | **0.1** | 695 |  | 345 | **0.1** |  | 365 | **0.1** |  | 710 |
| Asthma and COPD | 0 | 480440 | **90.7** |  | 86815 | **90.2** | 567255 |  | 382825 | **90.8** |  | 184400 | **90.2** | 567225 |  | 273080 | **90.8** |  | 294145 | **90.3** |  | 567225 |
|  | 1 | 49460 | **9.3** |  | 9465 | **9.8** | 58925 |  | 38870 | **9.2** |  | 20090 | **9.8** | 58960 |  | 27520 | **9.2** |  | 31435 | **9.7** |  | 58955 |
| Heart failure | 0 | 529740 | **100.0** |  | 96235 | **100.0** | 625975 |  | 421555 | **100.0** |  | 204415 | **100.0** | 625970 |  | 300495 | **100.0** |  | 325480 | **100.0** |  | 625975 |
|  | 1 | 165 | **0.0** |  | 45 | **0.0** | 210 |  | 135 | **0.0** |  | 70 | **0.0** | 210 |  | 110 | **0.0** |  | 100 | **0.0** |  | 210 |
| Connective tissue | 0 | 527240 | **99.5** |  | 95820 | **99.5** | 623060 |  | 419565 | **99.5** |  | 203490 | **99.5** | 623055 |  | 299095 | **99.5** |  | 323965 | **99.5** |  | 623065 |
|  | 1 | 2660 | **0.5** |  | 460 | **0.5** | 3125 |  | 2130 | **0.5** |  | 1000 | **0.5** | 3125 |  | 1505 | **0.5** |  | 1615 | **0.5** |  | 3120 |
| Dementia | 0 | 529355 | **99.9** |  | 96180 | **99.9** | 625535 |  | 421265 | **99.9** |  | 204270 | **99.9** | 625535 |  | 300290 | **99.9** |  | 325250 | **99.9** |  | 625540 |
|  | 1 | 545 | **0.1** |  | 100 | **0.1** | 645 |  | 430 | **0.1** |  | 220 | **0.1** | 650 |  | 310 | **0.1** |  | 330 | **0.1** |  | 640 |
| Diabetes | 0 | 525330 | **99.1** |  | 95400 | **99.1** | 620730 |  | 417995 | **99.1** |  | 202760 | **99.2** | 620750 |  | 297905 | **99.1** |  | 322825 | **99.2** |  | 620730 |
|  | 1 | 4570 | **0.9** |  | 880 | **0.9** | 5450 |  | 3700 | **0.9** |  | 1730 | **0.8** | 5430 |  | 2695 | **0.9** |  | 2755 | **0.8** |  | 5450 |
| Diabetes complications | 0 | 527555 | **99.6** |  | 95830 | **99.5** | 623385 |  | 419805 | **99.6** |  | 203580 | **99.6** | 623385 |  | 299245 | **99.5** |  | 324145 | **99.6** |  | 623390 |
|  | 2 | 2345 | **0.4** |  | 450 | **0.5** | 2795 |  | 1885 | **0.4** |  | 910 | **0.4** | 2795 |  | 1355 | **0.5** |  | 1435 | **0.4** |  | 2790 |
| Hemiplegia | 0 | 529800 | **100.0** |  | 96260 | **100.0** | 626060 |  | 421615 | **100.0** |  | 204445 | **100.0** | 626060 |  | 300540 | **100.0** |  | 325520 | **100.0** |  | 626060 |
|  | 2 | 100 | **0.0** |  | 20 | **0.0** | 120 |  | 80 | **0.0** |  | 45 | **0.0** | 120 |  | 60 | **0.0** |  | 60 | **0.0** |  | 120 |
| HIV | 0 | 528350 | **99.7** |  | 95755 | **99.5** | 624105 |  | 420410 | **99.7** |  | 203655 | **99.6** | 624065 |  | 299605 | **99.7** |  | 324465 | **99.7** |  | 624070 |
|  | 6 | 1555 | **0.3** |  | 525 | **0.5** | 2080 |  | 1285 | **0.3** |  | 830 | **0.4** | 2115 |  | 1000 | **0.3** |  | 1115 | **0.3** |  | 2110 |
| Metastatic cancer | 0 | 529870 | **100.0** |  | 96270 | **100.0** | 626140 |  | 421665 | **100.0** |  | 204475 | **100.0** | 626140 |  | 300580 | **100.0** |  | 325560 | **100.0** |  | 626140 |
|  | 6 | 30 | **0.0** |  | 10 | **0.0** | 40 |  | 25 | **0.0** |  | 15 | **0.0** | 40 |  | 20 | **0.0** |  | 20 | **0.0** |  | 40 |
| Mild liver | 0 | 529470 | **99.9** |  | 96195 | **99.9** | 625665 |  | 421340 | **99.9** |  | 204325 | **99.9** | 625665 |  | 300325 | **99.9** |  | 325340 | **99.9** |  | 625665 |
|  | 1 | 430 | **0.1** |  | 85 | **0.1** | 515 |  | 350 | **0.1** |  | 165 | **0.1** | 515 |  | 280 | **0.1** |  | 240 | **0.1** |  | 515 |
| Mod-severe liver | 0 | 529865 | **100.0** |  | 96270 | **100.0** | 626130 |  | 421660 | **100.0** |  | 204470 | **100.0** | 626130 |  | 300575 | **100.0** |  | 325555 | **100.0** |  | 626130 |
|  | 3 | 40 | **0.0** |  | 10 | **0.0** | 50 |  | 30 | **0.0** |  | 20 | **0.0** | 50 |  | 25 | **0.0** |  | 25 | **0.0** |  | 50 |
| Mod-severe renal | 0 | 520760 | **98.3** |  | 94705 | **98.4** | 615465 |  | 414445 | **98.3** |  | 201035 | **98.3** | 615480 |  | 295250 | **98.2** |  | 320230 | **98.4** |  | 615485 |
|  | 2 | 9145 | **1.7** |  | 1575 | **1.6** | 10715 |  | 7250 | **1.7** |  | 3455 | **1.7** | 10700 |  | 5350 | **1.8** |  | 5350 | **1.6** |  | 10700 |
| MI | 0 | 529855 | **100.0** |  | 96270 | **100.0** | 626120 |  | 421645 | **100.0** |  | 204470 | **100.0** | 626120 |  | 300570 | **100.0** |  | 325550 | **100.0** |  | 626115 |
|  | 1 | 50 | **0.0** |  | 10 | **0.0** | 60 |  | 45 | **0.0** |  | 15 | **0.0** | 60 |  | 30 | **0.0** |  | 30 | **0.0** |  | 65 |
| Peptic ulcer | 0 | 529520 | **99.9** |  | 96200 | **99.9** | 625720 |  | 421390 | **99.9** |  | 204335 | **99.9** | 625720 |  | 300380 | **99.9** |  | 325345 | **99.9** |  | 625725 |
|  | 1 | 380 | **0.1** |  | 80 | **0.1** | 460 |  | 305 | **0.1** |  | 155 | **0.1** | 460 |  | 220 | **0.1** |  | 235 | **0.1** |  | 455 |
| Peripheral vascular | 0 | 529855 | **100.0** |  | 96270 | **100.0** | 626125 |  | 421650 | **100.0** |  | 204470 | **100.0** | 626120 |  | 300565 | **100.0** |  | 325555 | **100.0** |  | 626120 |
|  | 1 | 50 | **0.0** |  | 10 | **0.0** | 60 |  | 40 | **0.0** |  | 20 | **0.0** | 60 |  | 35 | **0.0** |  | 25 | **0.0** |  | 60 |

| Additional File 1- Tab.S2: Ten most frequent birth and postnatal codes within the study period | | |
| --- | --- | --- |
| Delivery code | **SNOMED code** | **Frequency** |
| Spontaneous vertex delivery | 309469004 | 81850 |
| Single live birth | 169826009 | 74640 |
| Delivery normal | 48782003 | 51125 |
| Elective caesarean section | 177141003 | 26820 |
| Emergency caesarean section | 274130007 | 26600 |
| Caesarean section | 11466000 | 25135 |
| Baby male | 169838000 | 24305 |
| Baby female | 169839008 | 22845 |
| Normal delivery procedure | 177184002 | 18655 |
| Birth of child | 169836001 | 14190 |
|  | | |
| Postnatal code | **SNOMED code** | **Frequency** |
| Maternal postnatal 6 week examination | 384636006 | 45670 |
| Postnatal examination normal | 169784003 | 28140 |
| Postnatal maternal examination | 384634009 | 13340 |
| Postpartum care | 133906008 | 8035 |
| Maternal postnatal examination done | 444136005 | 2075 |
| Full postnatal examination | 384635005 | 1875 |
| Notification of birth | 312486000 | 1585 |
| Postpartum depression | 58703003 | 1390 |
| Postnatal examination invitation | 880461000000109 | 1345 |
| Postnatal examination status | 243831005 | 1340 |
| *All counts were rounded to nearest five; data is representative of the eight-week follow-up cohort.* | | |

| **Additional File 1- Tab.S3:** Incident rate ratios (IRR) for the rate of postnatal checks. IRRs were estimated comparing pre-pandemic rates with the first national lock-down period modelled as an interruption in an interrupted time series analysis for COVID-19 with Newey-West SEs (lag-1 autocorrelation adjustment) | | | | |
| --- | --- | --- | --- | --- |
| **Follow-up Cohort** | **Term** | **IRR** | **95% Confidence Interval** | |
| 6 weeks | (Intercept) | 0.19 | 0.18 | 0.20 |
|  | times | 1.00 | 0.99 | 1.00 |
|  | **covid** | **0.87** | **0.83** | **0.91** |
|  | time.since | 1.01 | 1.00 | 1.01 |
| 8 weeks | (Intercept) | 0.37 | 0.35 | 0.39 |
|  | times | 0.99 | 0.99 | 1.00 |
|  | **covid** | **0.84** | **0.78** | **0.91** |
|  | time.since | 1.01 | 1.00 | 1.02 |
| 12 weeks | (Intercept) | 0.46 | 0.45 | 0.47 |
|  | times | 1.00 | 1.00 | 1.00 |
|  | **covid** | **1.06** | **1.02** | **1.10** |
|  | time.since | 1.01 | 1.00 | 1.01 |
| IRR for covid still demonstrate an immediate drop ~16% for 6- and 8- weeks, suggesting autocorrelation does not bias the point estimate. The confidence intervals are slightly wider which is expected as ignoring autocorrelation can underestimate standard errors. Pre-pandemic trend (times) still crosses 1, suggesting little change before the pandemic, and post-pandemic slope (time.since) IRR >1, showing recovery trend. | | | | |

| 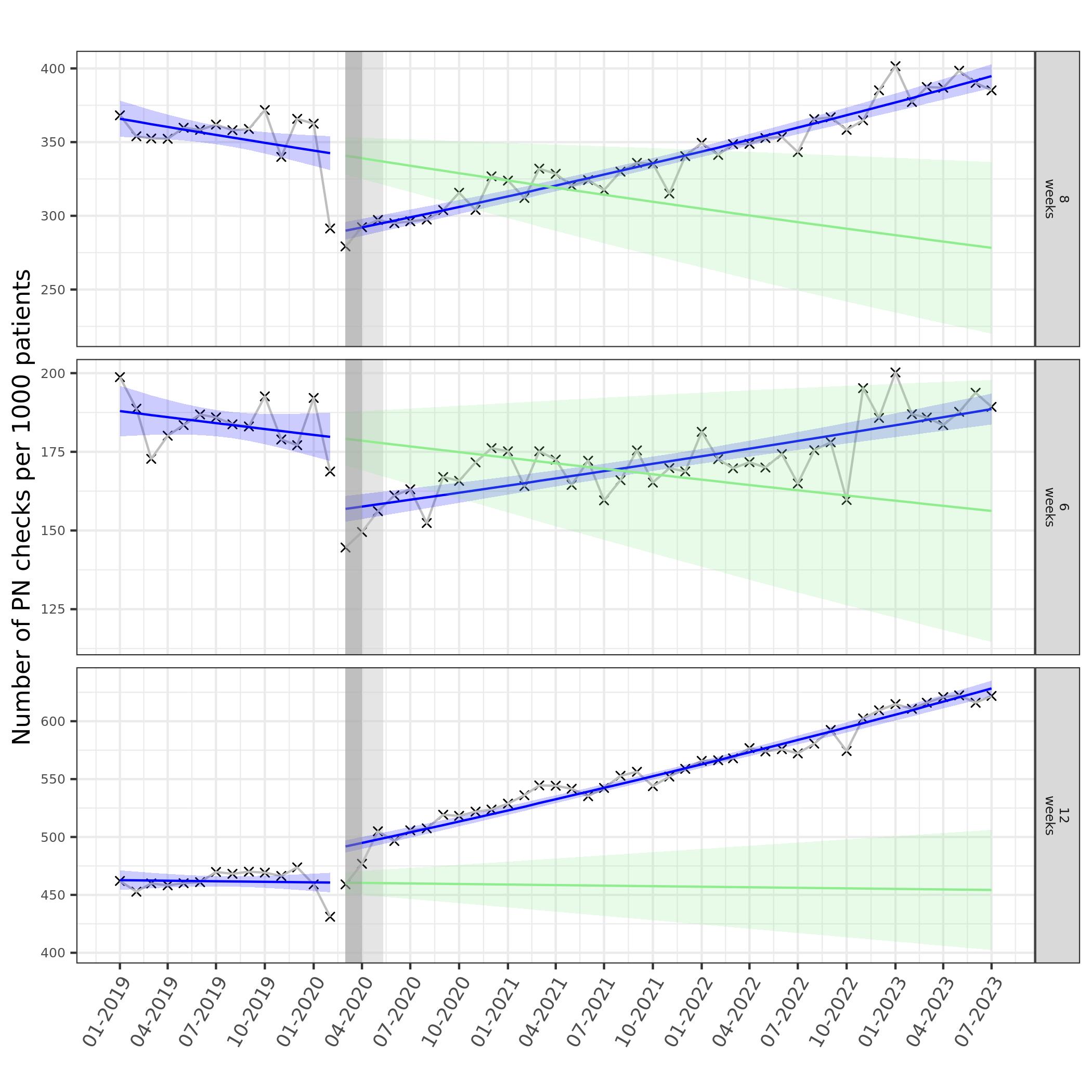 |
| --- |
| **Additional File 1- Fig.S1:** Interrupted Time Series analysis of the rate of postnatal examinations 6-, 8-, or 12-weeks follow-up cohorts. Negative binomial model (blue), Actual rate (black cross), Counterfactual modelled (green). |

| Additional File 1- Tab.4: Modelling the association of patient characteristics for no postnatal examination within eight weeks follow-up in 2019 compared to 2022. Data presented as odds ratio and 95% confidence intervals. | | | | | | | | |
| --- | --- | --- | --- | --- | --- | --- | --- | --- |
|  | **2019** | | | | **2022** | | | |
|  | **OR** | **95% CI** | | **P Value** | **OR** | **95% CI** | | **P Value** |
| (Intercept) | **1.72** | 1.52 | 1.95 | 0.00 | **1.64** | 1.49 | 1.81 | 0.00 |
| Age | **0.996** | 0.993 | 0.998 | 0.002 | 0.998 | 0.996 | 1.000 | 0.12 |
| BMI | 0.998 | 0.996 | 1.001 | 0.15 | **0.997** | 0.995 | 0.999 | 0.001 |
| Ethnicity |  |  |  |  |  |  |  |  |
| Asian or Asian British | **1.13** | 1.08 | 1.19 | 0.00 | **1.17** | 1.13 | 1.22 | 0.00 |
| Black or Black British | **1.21** | 1.12 | 1.32 | 0.00 | 1.06 | 0.99 | 1.13 | 0.08 |
| Chinese or Other Ethnic Groups | **1.25** | 1.13 | 1.37 | 0.00 | **1.16** | 1.08 | 1.25 | 0.00 |
| Mixed | **1.12** | 1.01 | 1.24 | 0.04 | **1.11** | 1.03 | 1.21 | 0.01 |
| Deprivation quintile |  |  |  |  |  |  |  |  |
| 1 | **1.40** | 1.34 | 1.47 | 0.00 | **1.28** | 1.23 | 1.33 | 0.00 |
| 2 | **1.19** | 1.14 | 1.24 | 0.00 | **1.16** | 1.12 | 1.21 | 0.00 |
| 3 | **1.11** | 1.06 | 1.16 | 0.00 | **1.07** | 1.03 | 1.11 | 0.00 |
| 4 | 1.03 | 0.98 | 1.08 | 0.24 | 1.01 | 0.98 | 1.05 | 0.48 |
| Region |  |  |  |  |  |  |  |  |
| East | **0.92** | 0.86 | 0.98 | 0.01 | 0.98 | 0.92 | 1.03 | 0.37 |
| East Midlands | **1.38** | 1.29 | 1.48 | 0.00 | **1.21** | 1.15 | 1.28 | 0.00 |
| North East | **1.51** | 1.39 | 1.65 | 0.00 | **1.50** | 1.39 | 1.61 | 0.00 |
| North West | **1.13** | 1.05 | 1.22 | 0.00 | **1.30** | 1.22 | 1.38 | 0.00 |
| South East | **0.71** | 0.65 | 0.76 | 0.00 | **0.93** | 0.87 | 0.996 | 0.04 |
| South West | **0.91** | 0.85 | 0.97 | 0.01 | **0.94** | 0.88 | 0.99 | 0.03 |
| West Midlands | **1.40** | 1.28 | 1.54 | 0.00 | **1.36** | 1.26 | 1.47 | 0.00 |
| Yorkshire and The Humber | **1.47** | 1.37 | 1.57 | 0.00 | **1.19** | 1.12 | 1.25 | 0.00 |
| Charlson comorbidity Index > 0 | 0.96 | 0.91 | 1.01 | 0.14 | 1.05 | 0.99 | 1.12 | 0.10 |
| When patients had more than one delivery code in the study period one was selected at random to model.  Reference categories were, Ethnicity: White, Deprivation quintile: 5 least deprived, Region: London, Charlson comorbidity index score of zero. | | | | | | | | |
